# Supplementary material for: Substrate and Fano Resonance Effects on the Reversal of Optical Binding Force between Plasmonic Cube Dimers
Source: Sci Rep. 2017 Jul 31;7:6938. doi: 10.1038/s41598-017-07158-z (PMC5537282; doi:10.1038/s41598-017-07158-z)
Supplement: Supplementary file 1 — Supplement article [file 41598_2017_7158_MOESM1_ESM.pdf]

Supplementary Information for "Substrate and Fano Resonance Effects on the Reversal of Optical Binding Force between Plasmonic Cube Dimers"

M.R.C. Mahdy*[1,2,3], Tianhang Zhang[2,4], Md. Danesh[2,5], Weiqiang Ding*[6]

[1]*Department of Electrical & Computer Engineering, North South University, Bashundhara, Dhaka 1229, Bangladesh*

[2]*Department of Electrical and Computer Engineering, National University of Singapore, 4 Engineering Drive 3, Singapore 117583*

[3]*Pi Labs Bangladesh LTD, ARA Bhaban,39, Kazi Nazrul Islam Avenue, Kawran Bazar, Dhaka, Bangladesh.*

[4]*NUS Graduate School for Integrative Sciences and Engineering, National University of Singapore, 28 Medical Drive, Singapore 117456*

[5]*Transcelestial Technologies, 32 Carpenter Street, Singapore 059911, Singapore*

[6]*Department of Physics, Harbin Institute of Technology, Harbin 150001, People's Republic of China*

[*] Corresponding authors: mahdy.chowdhury@northsouth.edu and wqding@hit.edu.cn

S1: Variation of inter-particle gap: Nature of binding force for 120 nm lengthened Silver cubes over Silver substrate

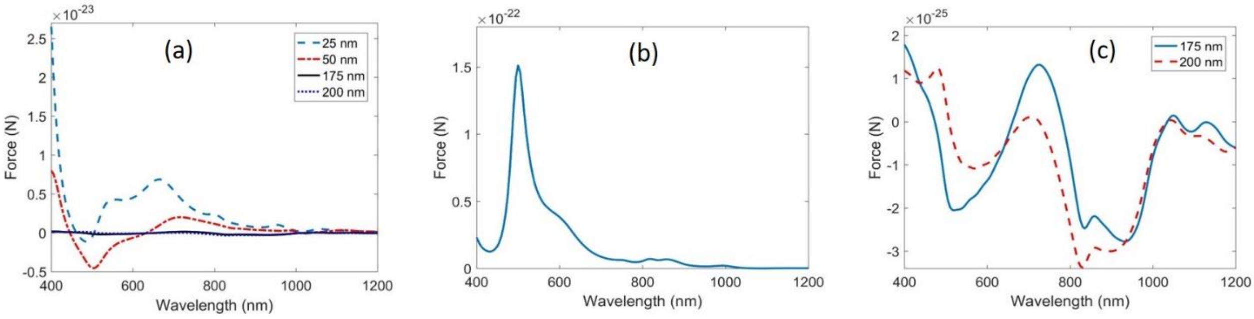

Fig.1s Optical binding force on two Ag cube homodimers (lengthened 120 nm) over Ag substrate with: (a) inter-particle gap of 25 nm, 50nm, 175 nm and 200 nm; (b) inter-particle gap of 10 nm and; (c) inter-particle gap of 175 nm and 200 nm.

For this specific full wave simulation with Ag homodimers, no spacer is used. Background medium is air. Cubes are placed 5nm (h= 5nm) away from the surface of silver substrate. *x*-polarized plane wave is propagating towards –*z*-direction.

S2: Binding force reversal for 180 nm lengthened Silver cubes over Silver substrate

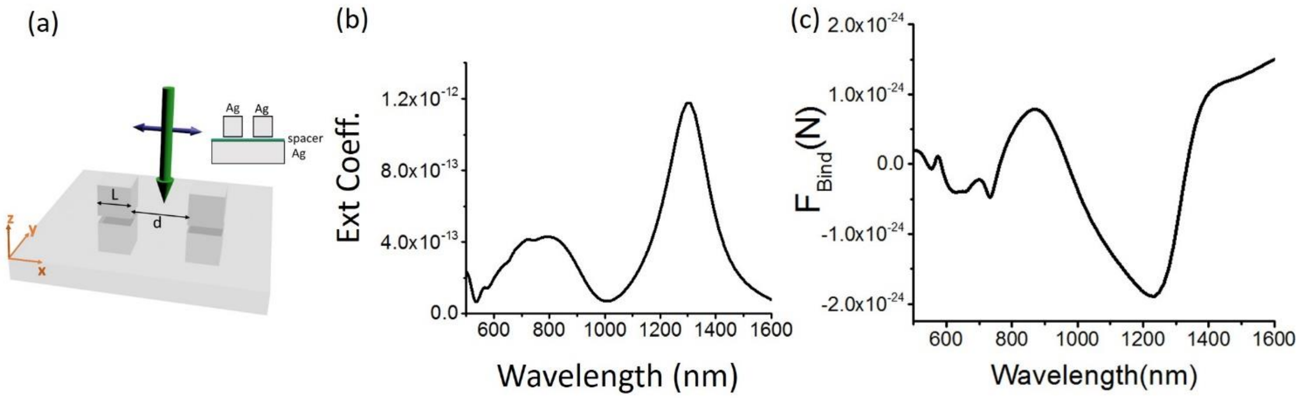

Fig.2s (a) Two silver cubes (L=180 nm) are placed over silver substrate and their inter-particle gap, *d*, is 150 nm. The spacer (refractive index 1.4) height is 5nm and background is water medium. Cubes are placed 5nm (h= 5nm) away from the spacer. *x*-polarized plane wave is propagating towards –*z*-direction. (b) Extinction co-efficient (c) Optical binding force.

S3: Binding force for heterodimer Silver cubes over Silver substrate

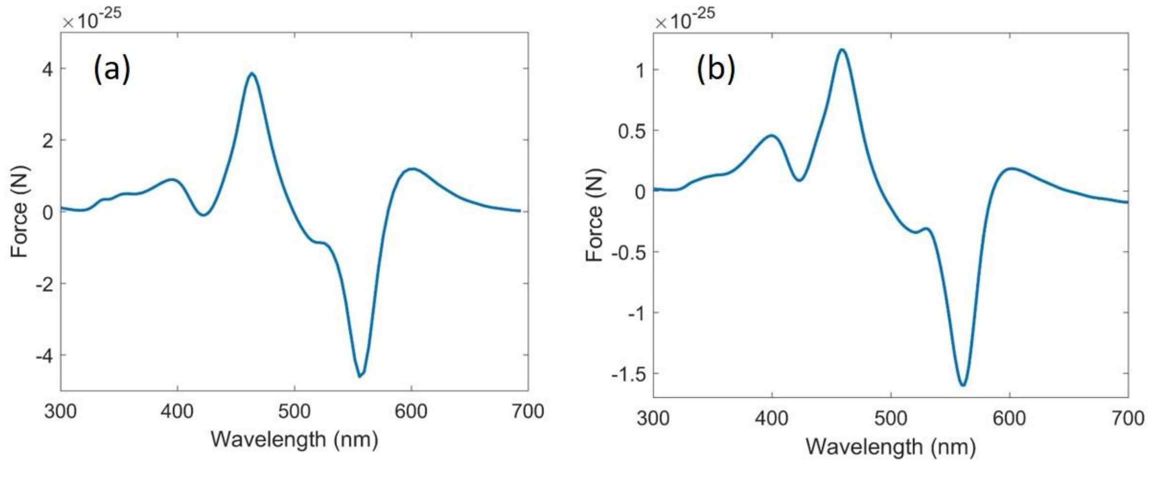

Fig. 3s Optical binding force on two Ag heterodimers (left one lengthened 60 nm and right one lengthened 120 nm; dimensions: $60\times60\times60\,nm^3$ and $120\times60\times60$ nm^3) over Ag substrate with: (a) inter-particle gap of 50nm and; (b) inter-particle gap of 100 nm.

For this specific full wave simulation with Ag heterodimers, no spacer is used. Background medium is air. Cubes are placed 5nm (h= 5nm) away from the surface of silver substrate. *x*-polarized plane wave is propagating towards –*z*-direction.

S4: Propagation of Surface plasmon polariton

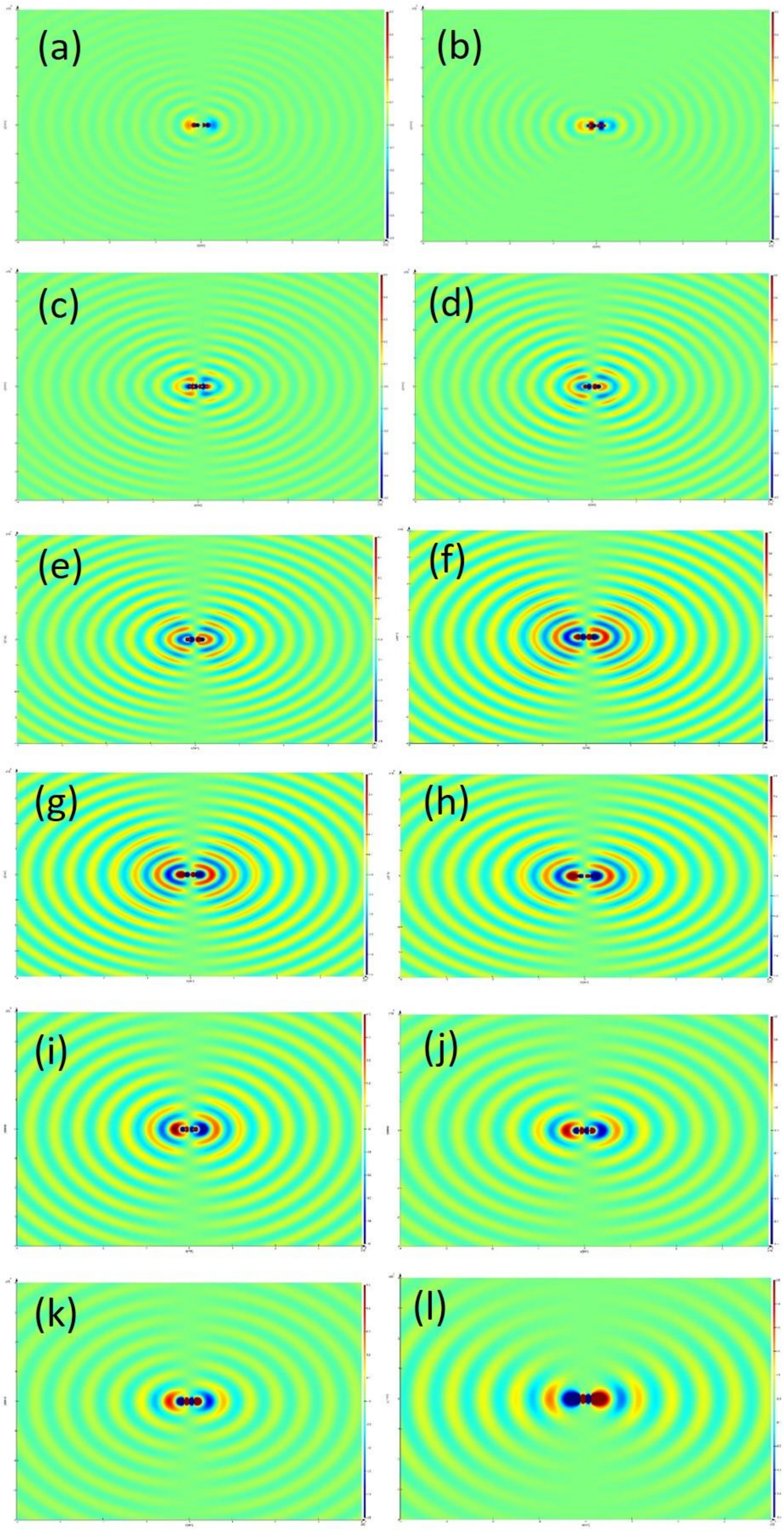

Fig.4s For 120 nm lengthened (inter-cube gap is 100 nm) cube homodimers over silver substrate, plot of weak propagation of weak surface plasmon polariton (SPP) at different wavelengths: (a) 550 nm (b) 570 nm (c) 590nm (d) 610 nm (e) 630 nm (around QQ resonance) (f) 682 nm (g) 710 nm (h) 740 nm (i) 773 nm (j) 810 nm (k) 848 nm (l) 966 nm (at DD resonance; SPP is much weaker in comparison with QQ resonance). Spacer height is always 5 nm and height of the homodimer cubes from the spacer is fixed 5nm from the spacer. Background is water medium.

S5: Binding force for homodimer Silver cubes over gold substrate

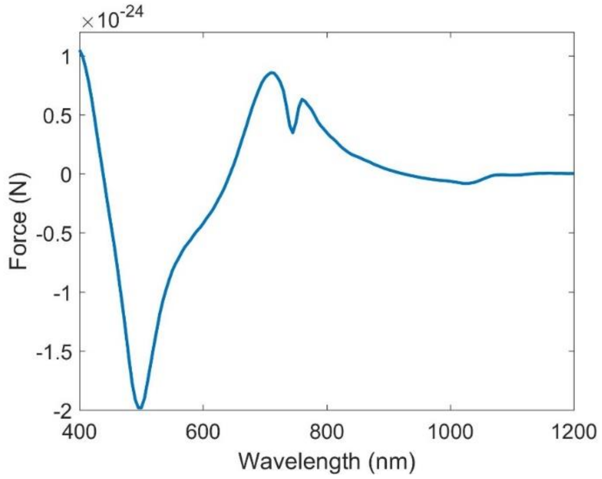

Fig. 5s Optical binding force on two Ag cube homodimers (lengthened 120 nm) over Au (gold) substrate with inter-particle gap of 100 nm.

For this specific full wave simulation with Ag homodimers, no spacer is used. Background medium is air. Cubes are placed 5nm (h= 5nm) away from the surface of silver substrate. *x*-polarized plane wave is propagating towards –*z*-direction.
